# A long-term study assessing the factors influencing survival and morbidity in the surgical management of bronchiectasis

**DOI:** 10.1186/1749-8090-6-161

**Published:** 2011-12-11

**Authors:** Abidin Sehitogullari, Salim Bilici, Fuat Sayir, Ufuk Cobanoglu, Ali Kahraman

**Affiliations:** 1 Van Training and Research Hospital, Department of Thoracic Surgery, Van-Turkey; 2 Yüzüncüyıl University, Faculty of Medicine, Department of Pediatric Surgery, Van-Turkey; 3 Yüzüncüyıl University, Faculty of Medicine, Department of Thoracic Surgery, Van-Turkey

**Keywords:** Bronchiectasis, surgical management, morbidity

## Abstract

**Background:**

Although the prevalence of bronchiectasis decreased significantly in developed countries, in less developed and in developing countries, it still represents a significant cause of morbidity and mortality. The aim of this retrospective study is to present our surgical experiences, the morbidity and mortality rates and outcome of surgical treatment for bronchiectasis.

**Methods:**

We reviewed the medical records of 129 patients who underwent surgical resection for bronchiectasis between April 2002 and April 2010, at Van Training and Research Hospital, Thoracic Surgery Department. Variables of age, sex, symptoms, etiology, and surgical procedures, mortality, morbidity and the result of surgical therapy were analyzed retrospectively.

**Results:**

Mean age was 21.8 year (the eldest was 67 year, the youngest was 4 years-old). Male/female ratio was 1.86 and 75% of all patients were young population under the age of 40. Bilateral involvement was 14.7%, left/right side ratio according to localization was 2.1/1. The most common reason for bronchiectasis was recurrent infection. Surgical indications were as follows: recurrent infection (54%), hemoptysis (35%), empyema (6%), and lung abscess (5%). There was no operative mortality. Complications occurred in 29 patients and the morbidity rate was 22.4%. Complete resection was achieved in 110 (85.2%) patients. Follow-up data were obtained for 123 (95%) of the patients. One patient died during follow-up. The mean follow-up of this patient was 9 months. Mean postoperative hospitalization time was 9.15 ± 6.25 days. Significantly better results were obtained in patients who had undergone a complete resection.

**Conclusions:**

Surgical treatment of bronchiectasis can be performed with acceptable morbidity and mortality at any age. The involved bronchiectatic sites should be resected completely for the optimum control of symptoms.

## Background

Bronchiectasis is defined as permanent dilatations of the bronchi with destruction of the bronchial wall. Although first described by Lannec at the beginning of the 19th century, it is still an important problem especially in some developing and underdeveloped countries [1]. Patients with early disease can be treated successfully by conservative measures. In those with disease involving multiple lobes of the lung or poor compliance with long-term medical treatment, resection has a definitive role in curing as well as improving the quality of life. The current increase in tuberculosis rates is directly related to insufficient and irregular medication. Additionally, irregular and inadequate treatment, the cessation of medication shortly after symptom improvement, and a lack of check-ups after treatment are factors accelerating recurrent pulmonary infection in developing countries. Therefore, bronchiectasis continues to be a major cause of morbidity and mortality in developing countries as well as in Turkey [2,3]. We reviewed the morbidity and mortality rates and outcome of surgical treatment for bronchiectasis.

## Methods

A retrospective chart review was conducted of 129 patients who underwent surgical resection for bronchiectasis between April 2002 and April 2010. The patients demographic features, the symptoms, etiologies and resection types, morbidity, mortality and outcomes after surgical management were analyzed. Patients were chosen as candidates for surgical treatment according to the following criteria: localized bronchiectasis documented by high-resolution computed tomography (HRCT), adequate cardiopulmonary reserve, symptoms such as chronic productive cough, repeated or significant hemoptysis, lung abscess, empyema, and recurrent pulmonary infections, and failure of medical treatment. Medical therapy constituted judicious use of systemic antibiotics based on current sputum or bronchoscopic lavage cultures, mucolytic agents, expectorants, postural drainage, humidification, anti-inflammatory agents, and bronchodilators. Fiberoptic bronchoscopy or rijit bronchoscopy was performed in all patients to rule out any intraluminal pathology. Failure of medical treatment was defined as frequent exacerbations interfering with normal professional and social life or requiring multiple hospitalizations. The focus of hemorrhage was identified bronchoscopically in patients with repeated or significant hemoptysis, and, after the lungs were assessed by HRCT, surgery was planned in patients who did not respond adequate to conservative treatment.

A double-lumen endotracheal tube was used in all patients to avoid a possible intraoperative contamination of contralateral lobes and for thoracic surgery. Pulmonary resection was achieved via a posterolateral thoracotomy. The decision for the volume of the resected lung was based on radiologic findings rather than on thoracotomy. All specimens had pathologic confirmation of bronchiectasis. Complete resection was defined as removal of all affected segments. The bronchial stump was closed using a mechanical stapler. The bronchial stump was not reinforced with any other tissue. The pulmonary resection was considered complete if the patient was believed to be free of bronchiectasis after thoracotomy. Operative mortality was defined as death within 30 days after thoracotomy. Chest physiotherapy was performed until discharge. All patients were given specific or wide-spectrum antibiotic therapy.

After surgery, patients were observed by a team that included chest physicians and surgeons. High-resolution CT examinations were performed once a year. Clinical data were calculated by the mean and standard deviation. The influences of some variables on the prognosis after the operation were studied by dividing the patients into an improved group ("excellent" or "good" outcomes) and an unimproved group ("no change" or "worse" outcomes). Logistic regression analyses were used to compare a variety of clinical factors between the groups. Additionally, the association between potential risk factors and postoperative complication was calculated. The parameters evaluated were patient demographics, symptoms, indications for surgery, choice of surgical procedure, morbidity, mortality, and functional outcome.

## Results

There were 84 (65.1%) male and 45 (34.8%) female patients; the average age of the 129 patients at thoracotomy was 21.8 years (range 4-67 years). The most common presenting symptoms were productive cough in 78 (60.4%) patients. There were 7 (5.4%) asymptomatic patients. Although these patients were asymptomatic at the time of admission, they had a history of recurrent pulmonary infections requiring medical therapy. The mean duration of the symptoms was 25.6 months (range, 1-135 months). One hundred two (79.00%) patients had chronic symptoms present for a mean of 41.2 months (range, 6-146 months). There was a history of tuberculosis in 18 (13.9%) patients. One hundred twenty-two (94.5%) patients had respiratory function tests, with the majority (79.5%) showing normal ventilatory patterns. The remainder showed a mixed or obstructive ventilatory pattern. The logistic regression analysis showed that a history of tuberculosis and incomplete resection were independent predictors of the operative result. Moreover, a forced expiratory volume in 1 second of less than 60% of the predicted value, a history of tuberculosis, and incomplete resection were independent predictors of postoperative complications.

Posteroanterior and lateral chest radiographs were first made as preoperative diagnostic studies in all patients. HRCT was performed in all patients (100%). V/Q scintigraphy was performed in 11 patients (8.5%). On V/Q scintigraphy, perfusion and ventilation were both impaired, but ventilation was more affected. We have been performing bronchoscopy on all patients preoperatively. The cause of secondary bronchiectasis in 8 of the patients were central obstruction or stenosis. We had 7 patients with paranasal sinusitis. These patients had nasal polyps due to chronic sinusitis. Before the operation, these cases were evaluated by a specialist, and 3 of these 7 patients underwent operation. Afterward, they were operated on for bronchiectasis. During their long follow-ups, they had no problems, whereas the other 4 cases had occasional infections

Preoperative medical treatment, which included appropriate antibiotic therapy and postural drainage, was given and supervised by chest physicians. Sputum was cultured for bacterial examination before surgery. Surgical treatment was considered if the medical treatment failed. We considered that the treatment was a failure if sputum production persisted despite several (3 or 4) courses of treatment or if there was a limited extent of disease on CT. As a rule, the extent of diseased lung to justify operation must be localized to achieve complete resection. The decision for the volume of the resected lung was based on radiologic findings rather than on thoracotomy.

Hundred twenty-one (93.7%) patients had respiratory function tests, with the majority (75.4%) showing normal ventilatory patterns. The remainder showed a mixed or obstructive ventilatory pattern. The 6-minute walk test was performed along a level hospital corridor in 8 younger children. Arterial oxygen saturation (SaO_2_) was measured by finger pulse oximetry before and during the test and monitored continuously. Measurements were recorded at 30-second intervals. All patients were able to walk continuously for the 6-minute period. The lowest SaO_2 _was used to calculate minimum SaO_2_. Mean minimum SaO_2 _was 94.1. Care was taken to ensure that SaO_2 _did not decrease below 90%. Along with exercise, a 2% or greater desaturation was considered to represent a risk [4]. Three patient seen to be at risk received 2 weeks of additional chest physiotherapy, incentive spirometry, and ambulation with physical therapy. The patient underwent surgery after this course of treatment. No patient was observed to have effort dyspnea after the test.

Sputum culture and sensitivity showed no bacterial growth in 61 (47.2%) patients, and mixed growth of mainly gram-negative organisms in the others: Haemophilus influenzae in 24 (18.6%), Escherichia coli and Klebsiella in 15 (11.6%), Pseudomonas in 6 (4.6%), and Streptococcus pneumoniae in 4 (3.1%). Sputum acid-fast bacillus was positive in 16 (12.4%) patients preoperatively; all were given antituberculous treatment and underwent surgery once the sputum was negative (Table 1). The most common probable etiology was postobstructive pneumonitis (Table 2). Pulmonary resection was performed failure of medical therapy in 70 (54%) patients, recurrent or massive hemoptysis in 45 (35%), lung abscess in 6 (5%), and empyema in 8 (6%).

**Table 1:** Microbiologic culture results of the patients

Microorganism	n	%
Haemophilus influenzae	24	18.6
asit-fast bacillus	16	12.4
Streptococcus pneumoniae	4	3.1
Pseudomonas aeruginosa	6	4.6
Bacteroides and other anaerobes	3	2.3
Klebsiella and Escherichia coli	15	11.6
Negative culture	61	47.2

**Table 2:** Etiologic factors of bronchiectasis

Etiology	n (%)
Postobstructive pneumonitis	28 (21.7%)
Pneumonia	21 (16.2%)
Tuberculosis	18 (13.9%)
Childhood infections	18 (13.9%)
Foreign body aspiration	5 (3.8%)
Pulmonary sequestration	1 (0.7%)
Unknown etiology	38 (29.4%)

Thoracotomy was performed in all patients. The disease was bilateral in 19 patients (14.7%) and mainly confined to the lower lobe in 72 (55.8%). The mean number of segments involved was 3.1 (range, one to 9 segments). Surgical treatment included lobectomy (lobectomy, lobectomy ± segmentectomy, lobectomy ± wedge resection) in 105 patients (81.3%), pneumonectomy in 8 (6.2%), wedge resection or segmentectomy in 16 (12.4%), and a combination of these approaches in 38 (29.4%). Disease was considered completely resected in 110 patients (85.2%) (Table 3).

**Table 3:** Surgical procedures in 129 patients with bronchiectasis

Procedure		No. of Patients
Pneumonectomy		
Left		5 (3.8%)
Right		3 (2.3%)
Lobectomy		
Right upper lobe		4 (3.1%)
Right middle lobe		13(10.0%)
Right lower lobe		11 (8.5%)
Left upper lobe		5 (3.8%)
Left lower lobe		40 (31.0%)
Lingulectomy or segmentectomy+ wedge resection	(bilateral)	6(4.6%)
Lobectomy ± segmentectomy (Bilateral) Bilobectomy		3 (2.3%)
Right upper lobe + right middle lobe		7 (5.4%)
Right middle lobe + right lower lobe		5 (3.8%)
Left upper lobe +	lingula	3 (2.3%)
Left lower lobe +	lingula	14 (10.8%)
Bilateral segmentectomy		10 (7.7%)

Of the 45 patients with hemoptysis, 3 had massive hemoptysis. These 3 patients underwent surgery after bronchoscopic evaluation and intervention. Massive hemoptysis was defined as expectorating more than 600 mL of blood in 24 hours. A rigid broncoscope was used under emergency conditions to determine the location of hemorrhage in patients with massive hemoptysis. Irrigation with physiologic saline with ice was performed to stop the bleeding. Then the bleeding bronchus was catheterized for 1 hour via a double-lumen endotracheal tube. In 2 of these 3 patients, the focus of hemorrhage was clearly detected; in the third the hem-orrhage could not be exactly localized. HRCT revealed signs of destroyed lung that required left pneumonectomy.

Complications occurred in 29 patients (22.4%) and included atelectasis requiring bronchoscopy in 7, a prolonged air leak (greater than 10 days) in 5, empyema in 5, pneumonia in 4, postoperative hemorrhage requiring reexploration in 3, arrhythmias in 2, postpneumonectomy pulmonary edema in 1, respiratory failure in 1, and bronchopleural fistula in 1 (Table 4). One patients (0.7%) who underwent pneumonectomy died 9 month later, The cause of death was respiratory failure in patients.

**Table 4:** Complications after surgery for bronchiectasis

Complication	No. of Patients
Bronchopleural fistula	1 (0.7%)
Postoperative bleeding	3 (2.3%)
Respiratory failure	1 (0.7%)
Empyema	5 (3.8%)
Prolonged air leak (> 10 day)	5 (3.8%)
Pulmonary edema	1 (0.7%)
Arrhythmias	2 (1.5%)
Atelectasis	7 (5.4%)
Pneumonia	4 (3.1%)
Total	29 (22.4%)

Follow-up was complete in 123 patients, with a mean of 5.3 years (range, 1 to 8 years). Overall, 74 patients (60.1%) were asymptomatic after pulmonary resection. Symptoms were improved and antibiotic requirements were decreased in 35 patients (28.4%). Fourteen patients (11.3%) had no improvement. In the group that had a complete resection, 105 patients were available for follow-up. Seventy (66.6%) were asymptomatic, 28 (26.6%) were improved, and 7 (6.6%) were unimproved. In the group that had an incomplete resection, 18 were available for follow-up. Four (22.2%) were asymptomatic, 7 (38.8%) were improved, and 7 (38.8%) were unimproved. Complete resection resulted in a significantly better result than incomplete resection (p < 0.05).

## Discussion

In developing countries, bronchiectasis is still common and is a significant major cause of morbidity and mortality [5]. As the disease progresses, physical activities become increasingly limited, patients fail to thrive, and ultimately they suffer from social deprivation, intrinsic depression, and respiratory failure. Although antibiotics and postural drainage are widely applied in the medical management of the disease, resection of the involved segment remains the only treatment modality that can offer a potential cure [6]. Proper treatment of early chest infections during childhood and improved health awareness may have contributed to the reduction of the incidence of bronchiectasis in the developed countries. In our series, a predisposing cause was identified in 16 patients (12.4%), predominantly previous tuberculosis infection; however, most cases remained idiopathic.

Chronic respiratory infections, especially if acquired in childhood, play an important role in the etiology of bronchiectasis. The majority of patients with bronchiectasis have a history of childhood infections or recurrent pneumonia, with rates as high as 77.7% reported in the literature [7]. In our series, 46 (35.6%) patients had postobstructive pneumonitis plus a history of pulmonary infection in childhood. Currently, it is understood that adequate treatment of childhood infections is the most important factor to prevent this irreversible disease. In the series Özdemir et al. [4]; developed bronchiectasis reported more frequently in low socioeconomic level in humans. Our series found a significant decrease in the number of patients who underwent surgical treatment for bronchiectasis over the years in our country. This decrease may be associated with improvements in health care. Bronchiectasis is usually caused by bronchopulmonary infection or bronchial obstruction; approximately half of patients can trace the onset of symptoms to a childhood infection. Primarily the symptoms are chronic cough and fetid sputum. In our series, Haemophilus influenzae and asit-fast bacillus were the most common organisms isolated from sputum.

The incidence and surgical importance of bronchiectasis has decreased in developed countries following the successful control of childhood infections [1,8]. But it remains surgically important in developing countries. We noted a marked reduction in the annual number of patients with bronchiectasis in our series. Despite advances in thoracic surgery, the optimal treatment for bronchiectasis remains controversial [3,9]. There is a broad consensus concerning the indications for surgical resection. Most physicians and surgeons reserve this treatment for localized bronchiectasis [10]. Multisegmental or bilateral bronchiectasis is generally regarded as a contraindication for surgery [11,12]. Nevertheless, we are confronted with cases of bronchiectasis involving multiple segments and lobes, with failure of conservative therapy, for which surgical intervention may be considered. In our study, 10 of 19 patients with bilateral bronchiectasis were successfully treated by staged thoracotomy.

Pulmonary function studies were performed in 122 (94.5%) of our patients. As was the case with other series [2,13,14], we found a normal ventilatory pattern in the majority of patients (79.5%). The remainder showed a mixed or obstructive ventilatory pattern. In 25 (14.1%) patients with low forced expiratory volume in 1 second (< 60% of the predicted value), the postoperative complication rate was significantly high. This indicated that surgery should be delayed in cases of severe inflammation until adequate control has been achieved. In addition, the preoperative treatment should include reducing airway obstruction and eliminating microorganisms from the lower respiratory tract, which consists of antimicrobial therapy, postural physiotherapy, bronchodilators, and corticosteroids [3].

The goals of surgical therapy for bronchiectasis are to improve the quality of life for those patients in whom medical treatment has failed and to resolve complications such as empyema, severe or recurrent hemoptysis, and lung abscess [3,15]. Complete and anatomic resection should be done with preservation of as much lung function as possible to avoid cardiorespiratory limitation [16]. It was reported that the symptoms persisted when incomplete resection was carried out [17]. We performed complete resection in 85.2% of our patients, and preoperative symptoms resolved completely in 67.0% and improved in 25.7% (92.7% benefited from the operation). In the light of these findings, we suggest that complete resection should be per-formed for the surgical treatment of bronchiectasis and that incomplete resection should only be used for the palliative treatment of certain life-threatening symptoms.

Postoperative complications were observed in 12.7% of patients who underwent complete resection and in 79% of those who underwent incomplete resection. When suggestive lung regions are not excised, with the aim of sparing as much lung tissue as possible, more postoperative complications occur and a second operation that carries a higher morbidity and mortality might be required to remove the residual diseased tissues [18]. Therefore, we suggest that, during intraoperative examinations, if suggestive areas that could not be determined by radiologic examination are present, these parenchymal areas should be resected to perform complete resection.

Complications usually range between 9.4% and 24.6% [10,15,19]. We reported 22.4% complication rate, mainly related to bleeding and prolonged air leak, emphasizing the need for good hemostasis and careful dissection. In our cases of mixed bilateral localized bronchiectasis, most of the patients had improvement with low morbidity after resection of the localized areas. In cases of bilateral cystic bronchiectasis, the role of surgery is limited to life-threatening complications as a palliative measure and such patients should be listed for lung transplantation if respiratory failure occurs [20].

Complete resection of bronchiectatic parenchyma while saving as much normal lung parenchyma as possible is the mainstay of surgical treatment. This aim can be achieved by lobectomy in most patients, but other resection types, such as wedge or segmental resection, can also be used in less affected areas. In the series of Dogan et al. [16], 41.5% of patients underwent lobectomy, 4.7% had a bilobectomy, and 14.8% underwent lobectomy + wedge resection. In the series of Agasthian et al. [3], lobectomy was the commonest type of resection with a rate of 64.2% (n = 134). In general, bronchiectasis affects the most dependent lung areas such as the basal segments of the lower lobes, the middle lobe or lingula. In our series bronchiectasis was confined to only one lobe in 73 (56.6%) patients. Therefore lobectomy was the most common type of resection performed in our series. Pneumonectomy was done in 12 patients, predominantly as expected on the left side because of the anatomy of left bronchus syndrome [21]. In our series was similar that in the literature.

At follow-up, 7 (38.8%) patients were found to have no change or worse symptoms than preoperatively, of whom 18 (94.7%) had incomplet resection; however, 93.3% had complet resection in the group with improved symptoms. Previous studies have shown various causes for persistent symptoms after resection [22,23]. In our series; Relations between clinical variables and operative results as follows; the p-value for duration of symptoms was 0,506, for a positive culture was 0,135, for FEV 1 was 0,205, for complete/incomplete resection was 0.000. In patients who undergoing complete resection operative results were significantly better, and less morbidity.

Follow-up was complete in 123 (95.3%) patients with a mean of 5.3 years (range, 1 to 8 years). Seventy-four (60.1%) patients were free of symptoms after surgical treatment, symptoms were improved in 35 (28.1%), and were unchanged or worse in 14 (11.3%). Of the patients who underwent complete resection, 93.2% benefited from the operation and, of those who underwent incomplete resection, only 61.1% benefited. The logistic regression analysis showed that tuberculosis history and incomplete resection were independent predictors of the none improvement group. Moreover, a forced expiratory volume in 1 second was less than 60% of the predicted value, tuberculosis history, and incomplete resection were independent predictors of postoperative complications (Tables 5).

**Table 5:** Relation between clinical variables and postoperative complications

Variables	OR	95%	CL	P Value
Gender (male:female),	0.584		0.248-1.376	.219
Age (< 17: > 17 ys)	1.926		0.728-5.108	.268
Symptoms duration (< 12: > 12 m)	1.256		0.465-3.392	.653
Positive culture (present:absent)	1.025		0.466-2.253	.951
FEV1(value < 60%: > 60 predicted)	0.164		0.059-0.458	.001
Type of resection	0.031		0.010-0.096	.000
(complete: incomplete)				
Tuberculosis (presentabsent)	0.052		0.025-1.345	.001

OR, Odds ratio; CI, confidence interval; FEV_1_, forced expiratory volume in 1 second.

Pulmonary resection can be performed with acceptable morbidity and mortality rates in properly selected patients. The morbidity rate reported in the literature is around 25% [3.24]. Common complications include atelectasis, hemorrhage, empyema, respiratory failure and bronchopleural fistula. The complication rate in our series was 22.4%, similar to that in the literature. Reported mortality rates in the literature range between 1 and 3% [3]. Our mortality rate was 0.7% which can be explained by the young age of our patient population (The mean follow-up of this patient was 9 months).

## Conclusions

Pulmonary resection for bronchiectasis can be done with low mortality and morbidity. The involved bronchiectatic sites should be resected completely for the optimum control of symptoms.

## Competing interests

The authors declare that they have no competing interests.

## Authors' contributions

AS, SB, FS, UC and AK acted in coseption and design. SB and AK acted in data analysis and interpretion. AS, and FS acted in manuscript writing. AS and UC acted in revision of the article. All authors read and approved the final manuscript.
